# Supplementary material for: Type III effectors of symbiotic Rhizobia include diverse predicted transcriptional and post-transcriptional modulators
Source: PLoS Biol. 2026 Oct 8;24(10):e3004038. doi: 10.1371/journal.pbio.3004038 (PMC13667924; doi:10.1371/journal.pbio.3004038)
Supplement: Supplementary file 1 [file pbio.3004038.s019.docx]

# **Response to Reviewers**

**Manuscript:** PBIOLOGY-D-26-01216R1. Structural modeling uncovers diverse transcriptional and post-transcriptional modulators among type III effectors of symbiotic Rhizobia

**Authors:** Albin Teulet and Sebastian Schornack

**Editorial comments**

Reviewer 1 thinks that a subset of the key predictions (2-3 representative structural modules) should be confirmed by performing targeted experimental validation of their proposed functions, which will strengthen the computational framework. Based on their specific comments and following discussion with the Academic Editor, we think that validation of some of the main predictions should be required for publication in PLOS Biology.

**Response:** We thank the Editor for their careful handling of the manuscript and for the constructive tone of the evaluation. We would like to clarify the nature and scope of the study, as we believe this bears directly on the request for experimental validation. This work was conceived, conducted, and presented as a purely computational structural-proteomics study, a category that PLOS Biology publishes and for which PLOS' own editorial position holds that experimental validation, while valuable, is not a precondition for publication but should be referenced where possible.

We respectfully note that our central predictions are not unsupported. They are anchored throughout to independent, already-published experimental results. The most abundant module, SU1, is conserved in the gall-inducing effectors HsvB and HsvG of *Pantoea agglomerans*, which have been experimentally characterised as nucleus-localised, DNA-binding transcriptional activators (Nissan et al., 2006; Nissan et al., 2012), and our independently predicted DNA-binding and activation regions map precisely onto their experimentally defined functional domains (Fig. 5G).

Likewise, the BPN domain (SU2) lies within the C-terminal region of ErnA that has been experimentally shown to localise to the plant nucleus and to associate with nucleic acids in planta (Teulet et al., 2019), and which carries the motifs experimentally required for nodule induction (Camuel et al., 2023; Haq et al., 2025; Fig. S10).

We therefore consider that the substance of the request to validate two to three representative modules is already met by the published literature incorporated into our analysis. *De novo* experimental validation of the remaining predictions is a distinct, multi-year experimental programme that we are actively planning but that cannot be completed within the revision window.

We have revised the manuscript to make its predictive, experimentally-anchored framing fully explicit rather than to append new wet-lab data, and we would welcome a decision on the manuscript as a Methods and Resources article with these revisions in place.

**Reviewer #1**

In this computational study, the authors performed a comprehensive bioinformatics analysis of type III effectors from symbiotic rhizobia using state-of-the-art structural prediction and comparative tools. They identified a modular architecture composed of 22 distinct structural units, several of which show similarity to known enzymatic domains or nucleic acid-binding folds. These findings suggest the existence of a previously unrecognized class of rhizobial effectors that may function as transcriptional or post-transcriptional regulators in plant hosts.

**Response:** We thank the reviewer for this accurate summary of the work and for recognising the analysis as comprehensive and based on state-of-the-art tools. We have ensured that the revised abstract and introduction state plainly that the modular architecture and the 22 structural units represent structure-based predictions of function, so that the computational and predictive nature of the study is unambiguous from the outset.

Overall, this is an informative and well-executed computational study that provides valuable insights into the structural and functional diversity of rhizobial effector proteins. However, a major limitation of the current work is the lack of experimental validation to support the predicted functions.

**Response:** We are grateful for the reviewer's positive assessment. We respectfully note, however, that the manuscript does incorporate experimental support for its principal predictions, drawn from prior peer-reviewed work, and we have made this support more prominent in the revised text.

The DNA-binding and transcriptional-activation function predicted for SU1, our most abundant unit, is supported by its conservation in the experimentally characterised transcriptional activators HsvB and HsvG of *Pantoea agglomerans*, with our predicted functional regions mapping onto their experimentally defined DNA-binding and activation domains (Fig. 5G; Nissan et al., 2006; Nissan et al., 2012). The nucleic-acid-binding function predicted for the BPN domain (SU2) is supported by ErnA, which has been experimentally shown to localise to the nucleus and to bind nucleic acids *in planta*, with the SU2-containing C-terminal region coinciding with motifs experimentally required for nodulation (Teulet et al., 2019; Camuel et al., 2023; Haq et al., 2025; Fig. S10).

The RdRP-like SU6 retains the experimentally defined catalytic aspartate essential for polymerase activity (Fig. 2B, Fig. S8), and our segmentation pipeline recaptures the crystallographically determined folds of all ten sequence-annotated units (RMSD < 3 A; TM-score >= 0.5; Fig. S5).

We have added a dedicated passage to the Discussion that consolidates these existing experimental anchors so that the supporting evidence is unmistakable to the reader.

To strengthen the biological relevance of the study, the authors are strongly encouraged to prioritize a subset of key predictions, ideally 2-3 representative structural modules, and perform targeted experimental validation of their proposed functions. Such validation would substantially boost confidence in the computational framework and significantly elevate the overall impact of the work.

**Response:** We fully agree that targeted experimental validation is the appropriate next step scientifically, and it forms the core of our planned follow-up work. We would respectfully make two points. First, for two of the most representative modules, SU1 and SU2, independent experimental validation of the predicted function already exists in the published literature, as described above, and is incorporated into the manuscript. This addresses the substance of your request to validate two to three representative modules. Second, generating new experimental data for the remaining predictions, for example *in vitro* DNA and RNA-binding assays, *in planta* identification of transcriptional targets, RdRP activity assays, and nodulation phenotyping of structure-guided domain mutants, requires assays and genetic systems that constitute a separate, multi-year experimental programme rather than a revision-scale addition. We are committed to this work and have begun planning it, but it cannot be completed within the revision period, and appending a partial version of it would change the nature and scope of the paper.

**Action:** We have revised the manuscript so that every functional assignment is described as a structure-based prediction or hypothesis, with the existing experimental anchors clearly flagged and the outstanding tests set out as explicit, falsifiable predictions for future work.

**Reviewer #2**

This is a well-conceived, well-executed, and well-presented study that changes the way we think about effectors in bacterial mutualisms with plants. The results point to a radically new set of effector functions related to regulation of host gene and protein expression. This paper will be of broad interest, and it will be catalytic, driving new inquiry centered on effector-dependent changes to the plant transcriptome, DNA and RNA targets of effectors, and use of the effectors for biotechnology. The authors have made available on Zenodo their complete set of structural models, and this will expedite such inquiry. The study is purely computational, but even without experimental data, the results are compelling and exciting. In one important case (SU1) claims are strongly supported by homology to functionally characterized effectors in other plant-associated bacteria (see Fig. 5 especially), providing not only support for the predicted function of SU1 but also support for the overall approach. The paper is well-suited to PLOS Biology and should be published without delay. I recommend only two minor edits, to the title and abstract, for consistency with the main text, and these could be made at proof stage:

**Response:** We are very grateful to the reviewer for this generous and insightful assessment, and in particular for recognising that the SU1 claims are strongly supported by homology to functionally characterised effectors in other plant-associated bacteria, which provides support both for the predicted function of SU1 and for the overall approach. This is precisely the kind of independent experimental anchoring described in our responses above.

We are also glad the reviewer values that we made the complete set of structural models on Zenodo available as a resource that will expedite further inquiry.

We have implemented both requested edits, which align the language of the title and abstract with the computational and predictive nature of the study, and we have propagated the same calibrated wording throughout the main text wherever functional inferences are described.

TITLE: insert "predicted" after "diverse"

**Response:** Accepted. The revised title reads: "Structural modeling uncovers diverse predicted transcriptional and post-transcriptional modulators among type III effectors of symbiotic Rhizobia."

ABSTRACT: Change "Our findings demonstrate" to "Our findings strongly suggest"; in the sentence above that, change "suggesting" to "pointing to"

**Response:** Accepted. The revised abstract reads: "...we discovered the BPN (B3 and PUA-like nucleic acid binding) domain as a structural mimic of plant B3-domain transcription factors, pointing to a direct mechanism for hijacking legume development. Our findings strongly suggest that rhizobia employ a modular domain-fusion strategy to act as direct genetic modulators, uncovering a conserved mechanism used by both symbionts and pathogens to hijack host developmental programs."

We have applied the same predictive phrasing consistently across the main text where functional inferences are presented.
